# Enhanced Monofocal Intraocular Lenses in Fuchs’ Endothelial Dystrophy Patients: Results from Triple Descemet Membrane Endothelial Keratoplasty Procedure

**DOI:** 10.3390/life14020243

**Published:** 2024-02-09

**Authors:** Rita Mencucci, Giovanni Romualdi, Chiara De Vitto, Michela Cennamo

**Affiliations:** Eye Clinic, Department of Neuroscience, Psychology, Pharmacology and Child Health (NEUROFARBA), University of Florence, Largo Brambilla 3, 50134 Florence, Italy; giovanni.romualdi@unifi.it (G.R.); devittochiara@gmail.com (C.D.V.); michela.cennamo@unifi.it (M.C.)

**Keywords:** cataract surgery, Fuchs disease, DMEK, monofocal intraocular lens, monofocal with enhanced intermediate vision lens, ocular aberration

## Abstract

Purpose: Descemet membrane endothelial keratoplasty (DMEK) is currently regarded as the most effective surgical procedure for addressing Fuchs Endothelial Corneal Dystrophy (FECD), frequently performed in conjunction with cataract surgery. In this retrospective study, we present a comparison of visual performance, clinical outcomes, and optical quality between two types of monofocal Intraocular Lenses (IOLs): one standard and one enhanced intermediate vision model, implanted in patients who underwent combined phacoemulsification and DMEK surgery. Methods: This single center comparative retrospective study was conducted at the Eye Clinic of the University of Florence (Italy) and included a total of 48 eyes of 48 patients affected by FECD and cataract. All patients underwent combined DMEK with phacoemulsification procedures. The patients’ data were analyzed and divided into two groups: one group (standard group) consisted of 24 eyes that underwent phaco-DMEK with implantation of a monofocal IOL, and the second group (enhanced monofocal group) included 24 eyes that underwent phaco-DMEK with implantation of an enhanced monofocal IOL. In both groups, the following monocular visual outcomes were evaluated 6 months after surgery: Uncorrected Distance Visual Acuity (UDVA) and Best-Corrected Distance Visual Acuity (BCDVA) at 4 mts; Uncorrected Intermediate Visual Acuity (UIVA), Distance-Corrected Intermediate Visual Acuity (DCIVA) at 66 cm; Uncorrected Near Visual Acuity (UNVA) and Best Corrected Near Visual Acuity (BCNVA). Monocular defocus curves were also assessed. Furthermore, optical quality in terms of Contrast Sensitivity (CS) in photopic conditions, Higher-Order Aberrations (HOAs) at a pupil size of 5 mm. Modulation Transfer Function (MTF), Objective Scatter Index (OSI), and Strehl ratio, were also analyzed. A Patient-Reported Spectacle Independence Questionnaire was revised to evaluate spectacle independence outcomes. Results: the two groups did not exhibit statistically significant differences in terms of UDVA, BCDVA, UNVA and BCNVA, photopic CS, HOAs, OSI, Strehl ratio, and MTF. However, in the phaco-DMEK enhanced monofocal IOL group, significantly better results were observed in terms of UIVA and DCIVA as well as a different defocus curve profile at 1.50 D, providing better defocus results at intermediate distance compared with the ZCB00 IOL. Conclusion: In our study, we found that enhanced monofocal lens performed favorable visual outcomes, even in cases of FECD, compared to standard monofocal IOLs. Comparable optical quality observed in the Eyhance group could allow surgeons to consider these lenses as a viable option for selected patients with FECD.

## 1. Introduction

Corneal endothelial diseases, such as Fuchs Endothelial Corneal Dystrophy (FECD), are prevalent causes of visual impairment and serve as an important indication for endothelial corneal transplantation. Currently, it is estimated that approximately 300 million people over the age of 30 worldwide are affected by FECD. By 2050, that number is expected to increase to 415 million people. Epidemiological data on FECD suggest that FECD preferentially affects middle-aged people and thus coincides with the time when patients develop cataracts and require cataract surgery [1,2]. Consequently, as the global population continues to age, it has become increasingly common for patients to require treatment for cataracts and FECD [1,2]. Cataract surgery must be approached with caution as it can cause corneal decompensation in patients with FECD [1,2].

One of the most commonly employed methods to manage a simultaneous presence of cataract and FECD is the combined surgery consisting of Descemet Membrane Endothelial Keratoplasty (DMEK) and phacoemulsification, often referred to as “triple DMEK” [3].

While cataract surgery with traditional monofocal intraocular lens (IOL) implantation has shown excellent outcomes in terms of distance vision, patients frequently require corrective spectacles for near vision. However, given the current increasing prevalence of computer use and of younger patients undergoing cataract surgery, there is a growing demand for intermediate vision correction as well [3,4]. 

Consequently, there has been a growing interest in the IOLs that permit a relative independence from spectacles for intermediate distance, while minimizing unwanted side effects and enhancing certain aspects of quality of life [4,5,6].

The TECNIS Eyhance DIB00 (Johnson & Johnson Vision Care, Irvine, CA, USA) is a newly developed monofocal IOL that incorporates a higher-order aspheric anterior surface [7,8].

The purpose of our study was to evaluate the difference in terms of visual outcomes and optical quality between two IOLs implanted in FECD patients who underwent a combined triple procedure: the standard Tecnis ZCB00 IOL versus Eyhance DIB00 IOL (Johnson & Johnson Vision Care, Irvine, CA, USA).

## 2. Material and Methods

### 2.1. Study Design and Patients

This comparative retrospective study involved patients who underwent a mono-lateral triple procedure (DMEK and cataract surgery). The surgery was performed at the Eye Clinic of Careggi University hospital, Florence, Italy from February 2021 to January 2023 (protocol number Ethics Committee FA000459, ID study 24175).

The inclusion criteria for our retrospective analysis were: patients affected by FECD ranging from grade 2 to 5 according to the Krachmer scale preoperatively as well as a cataract grade greater than II, according to the Lens Opacities Classification System III (LOCS III); patients of >60 years and with a CDVA > 0.4logMAR. We excluded individuals with conditions such as preoperative irregular astigmatism (e.g., keratoconus), regular corneal astigmatism more than ±0.75 diopters (D), central corneal thickness greater than 700 microns, amblyopia, axial length 25.0 mm or more, diabetes mellitus with retinal involvement, history of ocular surgery (even refractive surgery), previous episodes of uveitis, acute eye disease or infectious facts, intraocular pressure equal to 24 mmHg or higher, history of glaucoma with reduced sensitivity of visual field. Treatment with alpha blockers that can cause floppy iris syndrome, pathological miosis, pseudo exfoliation syndrome, post-surgical refractive outcome exceeding ±0.50 D, macular/retinal disease revealed by pre-surgical macular Optical Coherence Tomography (OCT), and dilated fundoscopy were also excluded.

### 2.2. Visual Outcome Assessments

We evaluated the following monocular preoperative visual parameters: Uncorrected Distance Visual Acuity (UDVA) and Corrected Distance Visual Acuity (CDVA) analyzed in photopic conditions using 85 cd/m [2] illumination with 100% contrast and the Early Treatment Diabetic Retinopathy Study (ETDRS) charts at a distance of 4 mt. The refractive error of the patients as spherical equivalent and simulated keratometry (Sim-K) values, derived from the anterior corneal curvature, were also evaluated through the Sirius tomographer (Florence, Italy). Pre-operative Endothelial Corneal Density (ECD) was also reported in both groups.

The following monocular visual outcomes were evaluated 6 months after surgery: UDVA and CDVA at 4 mt, Uncorrected Intermediate Visual Acuity (UIVA) and Distance-Corrected Intermediate Visual acuity (DCIVA) at 66 cm; Uncorrected Near Visual Acuity (UNVA) and Best Corrected Near Visual Acuity (BCNVA) at 44 cm. Monocular defocus curves analyzed after 6 months were tested using defocusing lenses with a power range of +2.00 D to −2.50. D in 0.5 D steps.

### 2.3. Optical Quality Data

We evaluate optical quality results through the AcuTarget HD Analyzer, which is an Optical Quality Assessment System (OQAS) product based on double-pass technology.

The Optical Quality Analysis System (OQAS) is an innovative tool that provides physicians with critical information about visual function. This facilitates measurement of the combined effects of high levels of optical aberrations and reduced ocular tissue transparency on retinal image quality. This technique provides an opportunity to objectively assess the effects of loss of transparency in ocular tissues by analyzing images of light spots focused on the retina and collected on the retinal plane.

The device measures various parameters at 4.0 mm pupil size, including the Objective Scatter Index (OSI), the Modulation Transfer Function (MTF) cutoff, and the Point Spread Function (PSF), expressed as the Strehl ratio. The OSI is a measurement of intraocular scatter light. It calculates the amount of light on the periphery of the double-pass image in relation to the amount of light in the center, while the MTF is the ratio between the contrast of the image and the contrast of the object. The MTF cutoff is the spatial frequency at which the MTF falls to 0. The point spread function represents the quality response of an imaging system and is expressed as the Strehl ratio, with a value of 1 indicating a perfect optical system [9]. Total High Order Aberrations (Total HOAs) at 5 mm optical zones (Osiris, CSO, Florence, Italy) were also analyzed through the Root Mean Square (RMS) and values were obtained of total, corneal and internal HOA for a mesopic pupil.

Monocular Contrast Sensitivity (CS) under photopic conditions was also examined through the Optec 6500 Vision Tester (Stereo Optical Co., Inc., Chicago, IL, USA).

Subjects underwent a gridded CS test contained in a chart of visual acuity (CSO) from a distance of 4 m. Measurements were performed monocularly using optimal refractive correction and natural pupil dilation. This device allows for CS measurements at 1.5, 3, 6, 12, and 18 cycles per degree (cpd). Grating stimuli were presented as Gabor patches. A sinusoidal grating is represented by a circular plate tilted vertically or 15 degrees to the right or left. Each sinusoidal grating appears as a uniformly illuminated background (85 cd/m^2^) with an amplitude of 5.4° × 4.3°, including a central region of full contrast (3.9°) and a peripheral ring of mixed contrast. A half-Gaussian ramp was added to each stimulus to minimize edge effects. For each spatial frequency, the instrument software reduces the contrast of the grating in steps according to the Quick Estimate by Sequential Testing (QUEST) method without fixed steps. The initial stimulus value is determined by the experimenter’s mode of a priori knowledge (maximum likelihood) about the probability density function of the threshold across the population. The subjects’ answers are then used to create a new probability density function using Bayes’ law. The next stimulus is presented at the most likely new threshold. This device can produce a contrast range of 0.02 to 2.25 Log CS. This corresponds to a percentage between 0.56 and 96.5%, with a minimum step size of 0.01 Log CS. Once all spatial frequencies have been tested, the device software provides a graph with CSF measurements expressed as Log CS or contrast percentage.

Six months after surgery, the Patient-Reported Spectacle Independence Questionnaire (PRSIQ) [10] was used to verify the subjective presence of glare, halos, and spectacle independence.

The PRSIQ serves as a tool to assess individuals’ independence from spectacles or contact lenses and gauges their satisfaction with vision across varying distances. Comprising 13 items, each offering 5 response options, ranging from 5 denoting “never/completely satisfied” to 1 indicating “all the time/dissatisfied”, the questionnaire is structured to address specific aspects. Items 1a to 1d focus on spectacle independence, considering the use of additional correction at different distances. Items 2a to 2d evaluate satisfaction with vision without additional correction, while items 3a to 3e appraise satisfaction with performance in different visual tasks [10].

### 2.4. Intraocular Lenses

The determination of IOL power and predicted postoperative refraction was based on the biometric data obtained through the IOL Master 500 (Zeiss, Oberkochen, Germany). We used the Barret Universal II formula for IOL power calculations. All implanted IOLs were carefully selected for each patient based on the biometric target closest to emmetropia.

### 2.5. Surgical Technique

The surgical procedure for both lenses was conducted in a similar manner by the same surgeon (RM). The standard surgical technique involved performing a one-injection block at the junction of the lateral one-third and the medial two-thirds of the infraorbital rim. This was achieved using a semi-sharp 25-gauge, 3.8 cm needle. A syringe containing 4 mL of 0.75% ropivacaine, 4 mL of 2% mepivacaine, and 150 units of hyaluronidase was used for the injection.

All surgical procedures were performed by the experienced surgeon (RM) from February 2021 to January 2023. Posterior lamellar grafts, specifically pre-cut DMEK grafts, were provided by the Eye Bank of Lucca in Italy.

After standard phacoemulsification and accurate removal of cohesive OVD, to prevent the collapse of the AC, an anterior chamber maintainer (ACM) was utilized. The endothelium and Descemet membrane were then stripped from the central area of the cornea, ranging from 8.5 to 9 mm in diameter. This was achieved using an inverted Price-Sinskey hook along the epithelial reference line at an angle of approximately 45° or 90°. The removed flap was examined on the anterior surface of the patient’s cornea to ensure its integrity.

The DMEK surgery followed the “no-touch” technique. The trephined at 7.75–8 mm DMEK graft was carefully detached from the surrounding Descemet membrane, immersed in sterile balanced saline solution, and loaded into a specific injector (E. Janach S.R.L., Como, Italy) with a transparent glass cartridge. The rolled graft was then injected into the AC through the main incision, applying slow and continuous pressure. Subsequently, the graft was unfolded and positioned using techniques such as the Tap-tap technique and Dirisamer technique. After ensuring the correct orientation and centration, the graft was pressed against the recipient stroma by injecting air underneath.

Post-surgery, patients were instructed to maintain a supine position for at least 3 h. This was done to facilitate proper graft adherence and healing.

### 2.6. Statistical Analysis

SPSS Statistics for Windows, version 22.0 (IBM Corp, Armonk, NY, USA) was used for the statistical analysis. Kolmogorov–Smirnov tests were used to check the normality of the data distributions. T tests and Pearson chi-square tests were used to verify the differences between the 2 groups. For all cases, *p* < 0.05 was regarded as statistically significant. Data are presented as means Standard Deviation (SD).

## 3. Results

In our retrospective study, we considered a total of 48 eyes from 48 patients. Each group, the Eyhance DIB00 group, and the ZCB00 group consisted of 24 eyes of 24 patients who underwent combined DMEK and cataract surgery. Preoperative characteristics of the patients are presented in Table 1. There were no statistically significant differences between the two groups in terms of age, preoperative Spherical Equivalent (SE) (*p*-value = 0.991), Central Corneal Thickness (CCT) (*p*-value = 0.917), Endothelial Corneal Density (ECD) (*p*-value = 0.704), Uncorrected Distant Visual Acuity (UDVA) (*p*-value = 0.415), Corrected Distant Visual Acuity (CDVA) (*p*-value = 0.086), and preoperative keratometric cylinder and Axial Length (AL) (*p*-value = 0.851). Both groups had a mean rebubbling rate of 22% per eye.

### 3.1. Refractive and Visual Outcomes

Refractive and visual outcomes are summarized in Table 2. Six months post-op all patients reached good levels of UDVA and BCDVA (*p*-value = 0.102 and 0.103, respectively). The UIVA and the DCIVA were significantly better in the Eyhance DIB00 group compared to the ZCB group (*p*-value = 0.016 and 0.021, respectively).

### 3.2. Aberrometric Parameters

Post-operative analysis HOAs acquired at 5.0 mm pupil size are shown in Table 3. All parameters were less than 0.300 microns, with no significant differences in both groups. Spherical Aberration (SA), including Ocular (total SA), Corneal, and Internal, showed the same values for the two groups (*p*-value = 0.439, 0.349 and 0.572, respectively). There were no statistically significant differences between the two groups for any parameter.

### 3.3. Optical Quality

Table 3 shows the optical quality parameters using the OQAS at 4.0 mm pupil size. No significant differences between the two groups in terms of OSI, MTF, and Strehl ratio were found (*p*-value = 0.166, 0.063 and 0.062, respectively).

### 3.4. Monocular Defocus Curve

**Figure 1:**
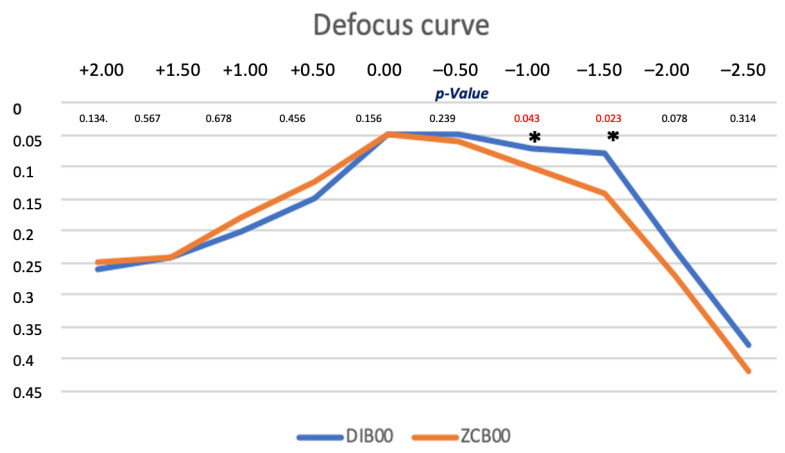
Comparison between mean defocus curves in the IOL groups (logMAR). * *p*-values for −1.00 D and −1.50 D, which are statistically significant (0.043 and 0.023).

The monocular defocus curve, as shown in Figure 1, reached best visual acuity at 0.00 D (4 m) in both groups, with a progressive reduction in visual acuity with the negative and positive defocus. While the ZCB00 IOL curve had an evident decrease with the addition of the negative defocus, the DIB00 IOL curve maintained a smoother profile, above all in correspondence with the intermediate defocus range (from −1 to −2 D), providing better defocus results at intermediate distance compared with the ZCB00 IOL. All *p*-values were >0.05, except for −1.00 and −1.50 D.

### 3.5. Contrast Sensitivity

**Figure 2:**
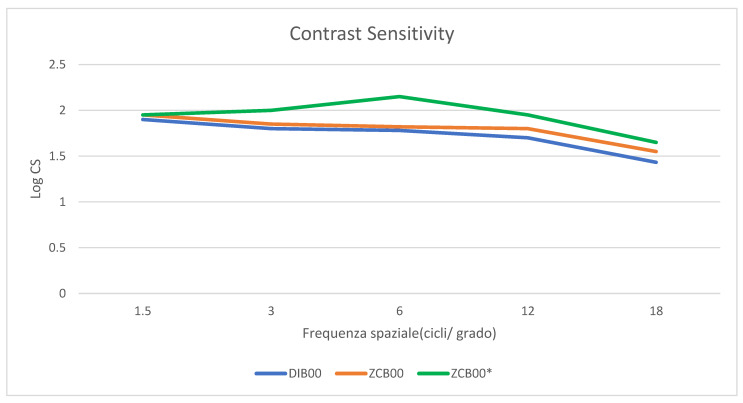
Monocular Contrast sensitivity measured with the Optec 6500 Vision Tester under photopic conditions at different spatial frequencies (cycles per degree) (LogCS = Log Contrast Sensitivity). * Green line represents the monocular CS curve under photopic condition of a single procedure of cataract surgery with standard monofocal IOLs, ZCB00 according to Mencucci et al.

The CS curve in photopic conditions is shown in Figure 2 and reveals no statistically significant differences in terms of photopic CS between the ZCB00 IOL and the Eyhance IOL for any spatial frequency.

### 3.6. Questionnaire

The findings from Table 4 present the outcomes of the Patient-Reported Spectacle Independence Questionnaire, which gauged the degree of autonomy from spectacles during daily life [10]. None of the patients indicated the need for spectacles to correct their distance vision. The DIB00 IOL group reported better independence from spectacles, particularly at intermediate distances, with only 23% of patients reporting a need for intermediate correction in their daily activities, compared to 87% of patients who had received the ZCB00 IOL. Another interesting result was highlighted from question three, in which the DIB00 group reported they were able to function comfortably at the intermediate distance without spectacles or contact lenses, in 70% of them all the time, unlike the ZCB00 group where no patient felt satisfied.

## 4. Discussion

FECD is a condition that affects the cornea, specifically the endothelial cells responsible for maintaining its clarity. When a patient with FECD also has cataract, it can be challenging for anterior segment surgeons to determine which condition is primarily responsible for the patient’s visual complaints. Cataract surgery itself may lead to the loss of endothelial cells and potentially worsen any existing corneal edema in FECD patients.

In recent times, there has been a growing trend and demand for addressing presbyopia during cataract surgery, and sometimes it can be a good choice even in patients with Fuchs endothelial corneal dystrophy [11]. Patients are increasingly interested in modern options such as bifocal, trifocal, or EDOF (Extended Depth Of Focus) IOLs, which provide excellent visual outcomes at various distances without the need for spectacles. The choice of a particular IOL depends on the patient’s individual characteristics, visual expectations, and preferences. Intermediate vision has gained significance due to the widespread use of computers, tablets, smartphones, and the need to read a car speedometer or navigate uneven terrain.

New IOL designs, such as trifocal or EDOF, have emerged to address the limitations in intermediate vision experienced with bifocal diffractive IOLs [12,13]. However, it is essential to consider that these advanced IOLs may cause halo or glare perception, which may not be well tolerated by all patients. Consequently, there is a continuous incentive to improve IOL technology and overcome these unwanted visual phenomena, leading to the development of the Eyhance DIB00 IOL. This monofocal IOL is specifically designed to enhance vision for intermediate tasks. The only difference lies in the modified aspherical anterior surface of the optics. This unique anterior surface aims to achieve a continuous power transition from the periphery to the center, creating a continuous power profile through the use of a higher-order asphere, ultimately improving intermediate vision. The DIB00 is based on refractive technology and does not incorporate diffractive rings or zones [14,15,16].

Previous studies showed that corneal HOA decreased after DMEK surgery in patients affected by FECD [17,18]. To the best of our knowledge, this is the first study that evaluates the performance of an enhanced intermediate monofocal lens in the triple DMEK procedure in patients with FECD.

This study yielded excellent UDVA and BCDVA outcomes for both types of IOLs. However, the Eyhance DIB00 group showed significantly higher UIVA and DCIVA at 66 cm. The Eyhance IOL also displayed a smoother defocus curve with a wider landing zone compared to the ZCB00 IOL.

Regarding HOAs, all parameters were not statical significant between Eyhance and the standard monofocal IOL groups, however, we noticed that the internal SA, expressed in RMS, was lower in the enhanced monofocal IOL group (−0.130 vs. −0.089 with a *p*-value of 0.378).

Despite the difference between the internal SA, the optical quality, measured using the OQAS, was similar in both groups concerning the OSI, MTF cutoff, and Strehl ratio. These optical quality parameters observed in the Eyhance group suggest that Enhanced monofocal IOLs do not affect qualitative visual outcomes in patients who underwent triple DMEK procedure. Previous studies confirmed that DMEK performs better in terms of quality of vision in comparison with UT-DSAEK [17]. Few studies have explored the optical quality in pseudophakic eyes using the OQAS.

Intermediate independence from spectacles, according to the PRSIQ, in the Eyhance group showed a good performance of this IOL, even after a lamellar transplant. Only 23% of DIB00-implanted patients needed spectacles for intermediate daily activities.

Additionally, as shown in a recent meta-analysis, the monocular CS was similar for both the Eyhance DIB00 and ZCB00 IOLs [19]. In our study the CS showed comparable results in both groups even after a DMEK triple procedure, although they were worse than for patients who underwent only cataract surgery implanted with enhanced monofocal IOLs.

The limits of our study are the design in terms of retrospective analysis and the post-operative criteria of selection that could affect the reproducibility of the results; moreover, glare and halometry analyses are missing. The importance of evaluating the halometry depends on the need to test glare disability, which can be considered one of the post-surgical subjective outcomes for the patient.

Other limitations could be the small sample size due to the relatively recent introduction of both the “Triple DMEK procedure” and enhanced monofocal IOLs and CS restricted to the only photopic condition, without having been tested in mesopic and scotopic conditions.

In conclusion, our retrospective study represents one of the first works where a triple procedure was performed using an enhanced monofocal lens. Our results show that the Tecnis Eyhance IOL may be a valid choice, not only for standard cataract treatment, but also as part of the triple procedure for selected patients with FECD. This IOL offers excellent freedom from glasses for intermediate tasks while maintaining visual quality comparable to the Tecnis ZCB00 one-piece monofocal IOL.

## Figures and Tables

**Table 1:** Preoperative parameters of patients in the 2 groups.

Assessments	Eyhance	ZCB	*p*-Value (ANOVA)
Age (mean ± SD) years	65.32 ± 2.12	65.08 ± 2.51	0.452
CCT (mean ± SD) micron	613.751 ± 46.572	610.003 ± 42.695	0.917
ECD (mean ± SD) cells/mm	1034.625 ± 73.858	1063.000 ± 76.963	0.704
UDVA (mean ± SD) logMAR	0.427 ± 0.081	0.432 ± 0.082	0.415
CDVA (mean ± SD) logMAR	0.382 ± 0.123	0.371 ± 0.103	0.086
SE (mean ± SD) D	1.662 ± 0.893	1.213 ± 0.732	0.991
CYL (mean ± SD) D	0.724 ± 0.202	0.702 ± 0.306	0.543
AL (mean ± SD) mm	23.586 ± 1.023	24.22 ± 0.683	0.851

CCT (Central Corneal Thickness); ECD (Endothelial Corneal Density); UDVA (Uncorrected Distant Visual Acuity), CDVA (Corrected Distant Visual Acuity), SE (Spherical Equivalent), CYL (Cylinder), AL (Axial Length).

**Table 2:** Six months post-operative monocular assessments (LogMar) of patients in the 2 IOL groups.

Assessments	Eyhance	ZCB	*p*-Value (ANOVA)
UDVA (mean ± SD)	0.072 ± 0.051	0.081 ± 0.053	0.102
BCDVA (mean ± SD)	0.04 ± 0.051	0.042 ± 0.052	0.103
UIVA (mean ± SD)	0.30 ± 0.100	0.40 ± 0.100	0.016 *
DCIVA (mean ± SD)	0.293 ± 0.090	0.370 ± 0.090	0.021 *
CIVA (mean ± SD)	0.071 ± 0.090	0.110 ± 0.090	0.909
UNVA (mean ± SD)	0.50 ± 0.105	0.542 ± 0.122	0.384
CNVA (mean ± SD)	0.08 ± 0.10	0.07 ± 0.08	0.473

Data are shown in logMAR (logarithm of the minimum angle of resolution); UDVA (Uncorrected Distant Visual Acuity), BCDVA (Best Corrected Distant Visual Acuity), UIVA (Uncorrected Intermediate Visual Acuity), DCIVA (Distant-Corrected Intermediate Visual Acuity), CIVA (Corrected Intermediate Visual Acuity), UNVA (Uncorrected Near Visual Acuity), CNVA (Corrected Near Visual Acuity). * *p* < 0.05 was regarded as statistically significant.

**Table 3:** Six months post-operative optical quality and aberrometric parameters.

Assessments	Eyhance	ZCB	*p*-Value (ANOVA)
OCULAR HOA RMS (micron)	0.266 ± 0.102	0.284 ± 0.104	0.439
CORNEAL HOA RMS (micron)	0.243 ± 0.055	0.246 ± 0.059	0.349
INTERNAL HOA RMS (micron)	0.225 ± 0.064	0.228 ± 0.071	0.572
OCULAR SA RMS (micron)	−0.055 ± 0.045	−0.021 ± 0.018	0.656
CORNEAL SA RMS (micron)	0.075 ± 0.075	0.068 ± 0.022	0.393
INTERNAL SA RMS (micron)	−0.130 ± 0.038	−0.089 ± 0.027	0.378
OSI	1.841 ± 0.0441	1.788 ± 0.272	0.166
MTF	24.994 ± 619.608	25.121 ± 671.979	0.063
PSF STREHL RATIO	0.152 ± 0.031	0.154 ± 0.028	0.062

HOA (High Order Aberration), SA (Spherical Aberration), OSI (Objective Scatter Index), MTF (Modulation Transfer Function).

**Table 4:** Patient-Reported Spectacle Independence Questionnaire (PRSIQ) results.

	Category 1 (%)		Category 2 (%)		Category 3 (%)		Category 4 (%)		Category 5 (%)	
	DIB00	ZCB00	DIB00	ZCB00	DIB00	ZCB00	DIB00	ZCB00	DIB00	ZCB00
(1) Did you need spectacles for:										
D	-	-	100%	100%	-	-	-	-	-	-
I	23%	87%	15%	8%	-	-	-	-	-	-
N	93%	97%	1%	-	-	-	-	-	-	-
(2) How often did you wear spectacles or contact lenses for:										
D	-	-	-	-	-	-	-	-	98%	100%
I	-	60%	6%	25%	5%	12%	11%	15%	75%	8%
N	38%	90%	19%	12%	21%	7%	11%	-	13%	-
(3) Were you able to function comfortably without spectacles or contact lenses for:										
D	100%	100%	-	-	-	-	-	-	-	-
I	70%	-	22%	10%	13%	20%	16%	20%	-	70%
N	-	-	7%	-	20%	7%	22%	7%	56%	90%

D = distance; I = intermediate; N = near. Wear and Function items used the verbal response categories “All of the time” (1), “Most of the time” (2), “Some of the time” (3), “A little of the time” (4), and “None of the time” (5).

## Data Availability

Data report is available in our Eye Clinic in our computer archive and the report is summarized for the publication.
